# Inhibition of Inwardly Rectifying Potassium (Kir) 4.1 Channels Facilitates Brain-Derived Neurotrophic Factor (BDNF) Expression in Astrocytes

**DOI:** 10.3389/fnmol.2017.00408

**Published:** 2017-12-07

**Authors:** Masato Kinboshi, Takahiro Mukai, Yuki Nagao, Yusuke Matsuba, Yoshimi Tsuji, Shiho Tanaka, Kentaro Tokudome, Saki Shimizu, Hidefumi Ito, Akio Ikeda, Atsushi Inanobe, Yoshihisa Kurachi, Seiji Inoue, Yukihiro Ohno

**Affiliations:** ^1^Laboratory of Pharmacology, Osaka University of Pharmaceutical Sciences, Osaka, Japan; ^2^Department of Neurology, Wakayama Medical University, Wakayama, Japan; ^3^Department of Epilepsy, Movement Disorders and Physiology, Graduate School of Medicine, Kyoto University, Kyoto, Japan; ^4^Department of Molecular and Cellular Pharmacology, Graduate School of Medicine, Osaka University, Osaka, Japan; ^5^Education and Research Center for Fundamental Pharmaceutical Sciences, Osaka University of Pharmaceutical Sciences, Osaka, Japan

**Keywords:** astrocytes, Kir4.1 channels, BDNF, epilepsy, antidepressants

## Abstract

Inwardly rectifying potassium (Kir) 4.1 channels in astrocytes regulate neuronal excitability by mediating spatial potassium buffering. Although dysfunction of astrocytic Kir4.1 channels is implicated in the development of epileptic seizures, the functional mechanisms of Kir4.1 channels in modulating epileptogenesis remain unknown. We herein evaluated the effects of Kir4.1 inhibition (blockade and knockdown) on expression of brain-derived neurotrophic factor (BDNF), a key modulator of epileptogenesis, in the primary cultures of mouse astrocytes. For blockade of Kir4.1 channels, we tested several antidepressant agents which reportedly bound to and blocked Kir4.1 channels in a subunit-specific manner. Treatment of astrocytes with fluoxetine enhanced BDNF mRNA expression in a concentration-dependent manner and increased the BDNF protein level. Other antidepressants (e.g., sertraline and imipramine) also increased the expression of BDNF mRNA with relative potencies similar to those for inhibition of Kir4.1 channels. In addition, suppression of Kir4.1 expression by the transfection of small interfering RNA (siRNA) targeting Kir4.1 significantly increased the mRNA and protein levels of BDNF. The BDNF induction by Kir4.1 siRNA transfection was suppressed by the MEK1/2 inhibitor U0126, but not by the p38 MAPK inhibitor SB202190 or the JNK inhibitor SP600125. The present results demonstrated that inhibition of Kir4.1 channels facilitates BDNF expression in astrocytes primarily by activating the Ras/Raf/MEK/ERK pathway, which may be linked to the development of epilepsy and other neuropsychiatric disorders.

## Introduction

Astrocytes are the major cell component of glial cells and play crucial roles in regulation of neural activity in the brain. By forming tripartite synapses in conjunction with presynaptic and postsynaptic neural components, astrocytes regulate ion and water homeostasis, metabolize neurotransmitters (e.g., glutamate, GABA, and glycine), and secrete various neuroactive substances including gliotransmitters, neurotrophic factors and cytokines (Araque et al., 1999; Parpura and Zorec, 2010; Devinsky et al., 2013). Among the diverse functions of astrocytes, spatial potassium buffering is a key mechanism for the maintenance of neural activity, which removes excessive extracellular K^+^ secreted from excited neurons (Walz, 2000; Kofuji and Newman, 2004; Simard and Nedergaard, 2004; Ohno et al., 2015). The potassium buffering currents are primarily mediated by the inwardly rectifying potassium (Kir) channels containing the Kir4.1 subunit, Kir4.1 channels (homo-tetramer of Kir4.1) and Kir4.1/5.1 channels (hetero-tetramer of Kir4.1 and Kir5.1), in astrocytes (Neusch et al., 2006; Ohno et al., 2015). In addition, spatial potassium buffering is known to be linked to glutamate and water uptake by astrocytes (Olsen and Sontheimer, 2008).

The Kir4.1 knockout animals showed severe motor impairment (e.g., ataxia and tremor), epileptic symptoms (e.g., jerky movements and convulsive seizures) and early mortality (within 3 weeks after birth) (Kofuji et al., 2000; Neusch et al., 2001; Djukic et al., 2007). In addition, Kir4.1 expression in astrocytes has been shown to be reduced (down-regulated) in the brain regions related to seizure foci in animal models of epilepsy (Ferraro et al., 2004; Inyushin et al., 2010; Harada et al., 2013). These findings suggest that dysfunction of astrocytic Kir4.1 channels can cause seizure generation probably by disrupting spatial potassium buffering. Furthermore, emerging evidence shows that loss-of-function mutations in the human *KCNJ10* gene encoding Kir4.1 cause the epileptic disorders called “EAST” (Epilepsy, Ataxia, Sensorineural deafness and Tubulopathy) or “SeSAME” (Seizures, Sensorineural deafness, Ataxia, Mental retardation, and Electrolyte imbalance) syndrome (Bockenhauer et al., 2009; Scholl et al., 2009; Reichold et al., 2010). Patients with EAST/SeSAME syndrome manifested a seizure phenotype characterized by generalized tonic-clonic seizures (GTCSs) with the initial appearance within a few months after birth. Moreover, association of single nucleotide polymorphisms (SNPs) of *KCNJ10* with temporal lobe epilepsy (TLE) accompanying febrile seizures (Heuser et al., 2010) and down-regulation of the Kir4.1 expression in seizure focus specimens from TLE patients (Das et al., 2012; Heuser et al., 2012; Steinhäuser et al., 2012) were also reported. Thus, dysfunction of Kir4.1 channels in astrocytes seems to be involved in the pathogenesis of human epileptic disorders. However, the pathophysiological mechanisms of astrocytic Kir4.1 channels in modulating epileptogenesis (development of chronic epilepsy) are still unknown.

Astrocytes release a variety of neuroactive molecules such as gliotransmitters (e.g., glutamate, ATP, and D-serine), neurotrophic factors [e.g., nerve growth factor (NGF), brain-derived neurotrophic factor (BDNF), and glia-derived neurotrophic factor (GDNF)], and cytokines [e.g., tumor necrosis factor-α (TNF-α) and interleukin-1β (IL-1β)] (Parpura and Zorec, 2010; Devinsky et al., 2013). Among them, BDNF is a key modulator of epileptogenesis, which influences on synaptic plasticity, neural sprouting, neurogenesis, and reactive gliosis (Kokaia et al., 1995; Jankowsky and Patterson, 2001; Hagihara et al., 2005; Jean et al., 2008; Quesseveur et al., 2013). Specifically, the mRNA and protein levels of BDNF are known to be elevated in various animal models of epilepsy and in human epileptic brains (Jankowsky and Patterson, 2001). In addition, genetic knockdown of BDNF has been shown to suppress the development of epilepsy (Kokaia et al., 1995; Barton and Shannon, 2005; Heinrich et al., 2011; Grabenstatter et al., 2012). Inhibition of the BDNF receptor TrkB has also been shown to prevent kindling epileptogenesis in TLE models and ameliorate status epilepticus (SE)-induced chronic recurrent seizures (Binder et al., 1999; Liu et al., 2013). Moreover, astrocytes express not only full-length TrkB, but also truncated TrkB receptors (Rose et al., 2003; Cheng et al., 2007), and the truncated TrkB, especially truncated TrkB-T1 receptors have been reported to facilitate astrogliosis, which may be involved in epileptogenesis (Goutan et al., 1998; Climent et al., 2000). All these evidences illustrate a causal role of BDNF in the development of epilepsy.

In the present study, we evaluated the effects of inhibition (blockade and knockdown) of Kir4.1 channels on the expression of BDNF and other neurotrophic factors in primary cultures of astrocytes to explore the mechanism of Kir4.1 channels in regulating epileptogenesis. For blockade of Kir4.1 channels, we tested several antidepressants agents (e.g., fluoxetine), which reportedly blocked Kir4.1 channels in a subunit-specific manner (Ohno et al., 2007; Su et al., 2007; Furutani et al., 2009; Ohno et al., 2015). In addition, small interfering RNA (siRNA) targeting Kir4.1 was employed to knockdown the Kir4.1 expression. The present results demonstrated for the first time that inhibition of Kir4.1 channels facilitates the expression of BDNF in astrocytes, which may be linked to the development of epilepsy and other neuropsychiatric disorders.

## Materials and Methods

### Astrocyte Primary Cultures

Primary cultures of cerebrocortical astrocytes were prepared from 1 or 2 day-old new born ICR mice (Japan SLC, Shizuoka, Japan). All the protocols of experiments were approved by the Animal Research Committee at Osaka University of Pharmaceutical Sciences. After brains were removed from the skulls under isoflurane anesthesia, meninges were excised carefully and forebrains were dissected into small pieces. Cells were dissociated with 0.25% trypsin (Sigma–Aldrich, St. Louis, MO, United States), plated to 25 cm^2^ flasks with Dulbecco’s Modified Eagle Medium (DMEM; Nacalai Tesque, Kyoto, Japan) containing 10% heat-inactivated fetal bovine serum (FBS; biowest, Nuaillé, France), and incubated at 37°C with 5% CO_2_/95% air. After the cells reached confluence (7 days), adherent cells were shaken by hand, detached with 0.05% trypsin, and plated to 75 cm^2^ flasks. After three times passage (21 days), the cells were plated to 24-well plates for mRNA analysis or 6 cm diameter dishes for protein analysis, and, thereafter, culture medium was replaced with serum-free DMEM. The cells were then subjected to the experiments under serum-free condition after 10 days or more.

### Immunofluorescence Staining

Astrocytes plated on glass coverslips were fixed in 4% paraformaldehyde, and then blocked in 0.3% Triton X-100 in phosphate buffered saline containing 1% bovine serum albumin (BSA; Wako, Osaka, Japan) for 30 min. After blocking, astrocytes were incubated with primary antibodies for glial fibrillary acidic protein (GFAP: a specific marker for astrocytes) (mouse monoclonal, 1:200; Progen, Heidelberg, Germany, RRID: AB_1541328), Kir4.1 (rabbit polyclonal, 1:200; Alomone Labs, Jerusalem, Israel, RRID: AB_2040120), BDNF (goat polyclonal, 1:200; Santa Cruz Biotechnology, Dallas, TX, United States, RRID: AB_2227954), and neuronal nuclear antigen (NeuN: a specific marker for neurons) (mouse monoclonal, 1:500; Millipore, Billerica, MA, United States, RRID: AB_2298772) at 4°C overnight. Subsequently, secondary antibodies of tetramethylrhodamine-5- (and 6)-isothiocyanate (TRITC; red fluorescence) goat anti-mouse (1:200; Sigma–Aldrich, RRID: AB_261699), fluorescein isothiocyanate (FITC; green fluorescence) goat anti-rabbit (1:200; Sigma–Aldrich, RRID: AB_259384), Alexa Fluor546 (red fluorescence) donkey anti-mouse (1:200; Thermo Fisher scientific, Waltham, MA, United States, RRID: AB_2534012), Alexa Fluor488 (green fluorescence) donkey anti-goat (1:200; Thermo Fisher scientific, RRID: AB_2534102), or Alexa Fluor647 (blue fluorescence) donkey anti-rabbit (1:200; Thermo Fisher scientific, RRID: AB_2536183) were, respectively, used for visualization. Immunofluorescence images were obtained with a confocal laser scanning microscope (Carl Zeiss Japan, LSM 700 ZEN, Tokyo, Japan).

### Quantitative Reverse Transcription (RT)-PCR

Total RNA was extracted from astrocytes in each well of a 24-well plate using ISOGEN^®^ (Nippon Gene, Tokyo, Japan) according to the manufacturer’s instructions. Purified total RNA was used to synthesize cDNA with a PrimeScript RT reagent Kit (Takara Bio, Shiga, Japan) for 15 min at 37°C and for 5 s at 85°C. cDNA was amplified in a PCR reaction containing SYBR^®^ Premix Ex Taq^®^ (Takara Bio) and 0.4 μM of each gene-specific primer (**Table 1**). The reactions were performed in a Thermal Cycler Dice Real Time System Single MRQ (Takara Bio; TP870) with a PCR profile comprising an initial denaturation step for 2 min at 95**°**C, 40 cycles with 5 s at 95**°**C and 60 s at 60°C, and a final step for 15 s at 95°C, 30 s at 60°C, and 15 s at 95°C. Following amplification, reaction specificity for each PCR run was confirmed by melt curve analysis.

**Table 1:** Sequences of siRNA and primers for quantitative reverse transcription (RT)-PCR.

	Sense strand (5′–3′)	Antisense strand (5′– 3′)
**Sequences of siRNA**		
Kir4.1	GCCAAGUUCGCACttcctacc	taggaaGUGCGAACUUGGCAG

	**Forward primer (5′–3′)**	**Reverse primer (5′–3′)**

**Sequences of primers for RT-PCR**		
GAPDH	TGTGTCCGTCGTGGATCTGA	TTGCTGTTGAAGTCGCAGGAG
Kir4.1	TGAGGCAATGCTGAGAGGAG	CCCGTAGGCAGAAAAGAGGA
BDNF	TCAAGTTGGAAGCCTGAATGAATG	CTGATGCTCAGGAACCCAGGA
GDNF	TCAGCTGCCCAGCACATTTC	TGGGAGCATCAGCTACCACATC
CNTF	GCTCACTTGTTTCCTGGGACAGT	CCATCCACTGAGTCAAGGCTGAT
NGF	TGCCAAGGACGCAGCTTTC	TGAAGTTTAGTCCAGTGGGCTTCAG
Slc6a4	TGGCTACATGGCTGAGATGAGG	AAGAATGTGGATGCTGGCATGTTA

### BDNF Enzyme-Linked Immunosorbent Assay (ELISA)

A quantitative measure of BDNF concentration in each cell lysate was performed with a BDNF E_max_^®^ ImmunoAssay System (Promega, Madison, WI, United States) according to the manufacturer’s instructions. As an internal control, total protein was measured in the lysates for use in normalizing results. The BDNF levels were expressed as a ratio to total protein.

### Kir4.1 siRNA Transfection

The sequences of siRNA targeting mouse Kir4.1 were designed as shown in **Table 1** (RNAi Co., Tokyo, Japan). Astrocytes were transfected with siRNA or a negative control using Lipofectamine^®^ RNAiMAX Transfection reagent (Thermo Fisher scientific) according to the manufacturer’s instructions. After 6 h of treatment with siRNA, the medium was replaced and incubated at 37°C with 5% CO_2_/95% air until collection.

### Western Blotting

Western blotting was performed as published previously (Ohno et al., 2009; Harada et al., 2013; Nagao et al., 2013). Briefly, cell lysates were incubated with sodium dodecyl sulfate-polyacrylamide gel electrophoresis (SDS-PAGE) sample buffer for 5 min at 95°C. Then, each sample (5 μg/lane) was run on a 15% polyacrylamide gel and transferred to a PVDF membrane (GE Healthcare, Buckinghamshire, United Kingdom). The membrane was blocked with TBS-T (25 mM Tris, 150 mM NaCl, and 0.1% Tween 20) containing 5% skimmed milk, and incubated with the primary antibodies for Kir4.1 (rabbit polyclonal, 1:500) or β-actin (mouse monoclonal, 1:1000; Sigma–Aldrich, RRID: AB_476743) at 4°C overnight. Then, the membrane was incubated with horseradish peroxidase (HRP)-conjugated secondary antibodies, goat anti-rabbit IgG-HRP conjugated (1:2000; Santa Cruz Biotechnology, RRID: AB_631746) or sheep anti-mouse IgG-HRP conjugated (1:2000; GE Healthcare, RRID: AB_772210) for 60 min at room temperature. Specific bands were detected with the enhanced chemiluminescence methodology (Amersham ECL Western blotting detection reagents and analysis system, GE Healthcare) by a lumino imaging analyzer (LAS-3000, FUJIFILM, Tokyo, Japan). To normalize for protein loading, the band intensity in each lane was standardized to the intensity of the β-actin band in the same lane after stripping and reprobing processes.

### Electrophysiology

Whole-cell patch clamp electrophysiology was performed as described previously (Ohno et al., 2007; Su et al., 2007). Briefly, human Kir4.1 inserted into IRES vectors (pIRES2-DsRed2, Clontech, Mountain view, CA, United States) were transfected into HEK293T cells using Fugene6 transfectant (Roche Diagnostics, Tokyo, Japan). The transfected cells were continuously perfused with a bathing solution (in mM; 112 NaCl, 30 KCl, 5 HEPES, 2 CaCl_2_, 0.53 MgCl_2_, and 5.5 glucose, pH7.4) at a flow rate of 1–1.5 ml/min. The recording micropipettes had input resistances of 1.4–1.8 MΩ when filled with an internal solution containing (in mM) 140 KCl, 2 MgCl_2_, 5 EGTA, and 5 HEPES (adjusted to pH7.25 with KOH). The cells were voltage-clamped at E_K_ (i.e., –40 mV in 30 mM [K^+^]_o_) and stepped to depolarized or hyperpolarized potentials (e.g., ± 70 mV) from E_K_. The clamp voltage and associated macroscopic currents were recorded with an Axopatch 200B amplifier (Axon Instruments, Union City, CA, United States), fed through a digitizer (Digidata 1322A, Axon Instruments) and stored in a computer with the data acquisition system Clampex 9.2 (Axon Instruments). Test drugs were dissolved in bathing solution and perfused until the response reached a plateau (usually 5 min). All experiments were performed at room temperature. In each cell, an excessive concentration (3 mM) of Ba^2+^ which totally blocked Kir channels was applied at the end of each experiment. The Kir channel currents were measured by subtracting the Ba^2+^-resistant currents from the total current. The response to the test drug was estimated as the current ratio (I_Drug_/I_Control_) at –110 mV, which was obtained by dividing the current recorded in the presence of the drug with the equivalent current recorded in the absence of the drug.

### Drugs

Sertraline hydrochloride, U0126, and SB202190 were purchased from Wako Pure Chemicals Industries (Osaka, Japan). Fluoxetine hydrochloride was purchased from LKT Labs (St. Paul, MN, United States). Imipramine hydrochloride and mianserin hydrochloride were purchased from Sigma–Aldrich. Fluvoxamine maleate and SP600125 were purchased from Tokyo Chemical Industries (Tokyo, Japan). Other common laboratory reagents were also obtained from commercial sources.

### Statistical Analysis

All data were expressed as the mean ± SEM. Comparisons between two groups were performed by Mann–Whitney test (mRNA levels or protein levels in astrocytes transfected with siRNA or exposed to each drug). Statistical significance of differences among multiple groups was determined by Kruskal–Wallis followed by Steel–Dwass *post hoc* test (mRNA levels in astrocytes exposed to multiple drugs or a certain drug at multiple concentrations). A *P*-value of less than 0.05 was considered statistically significant.

## Results

### Expression Patterns of Kir4.1 and BDNF in Cultured Astrocytes

**FIGURE 1:**
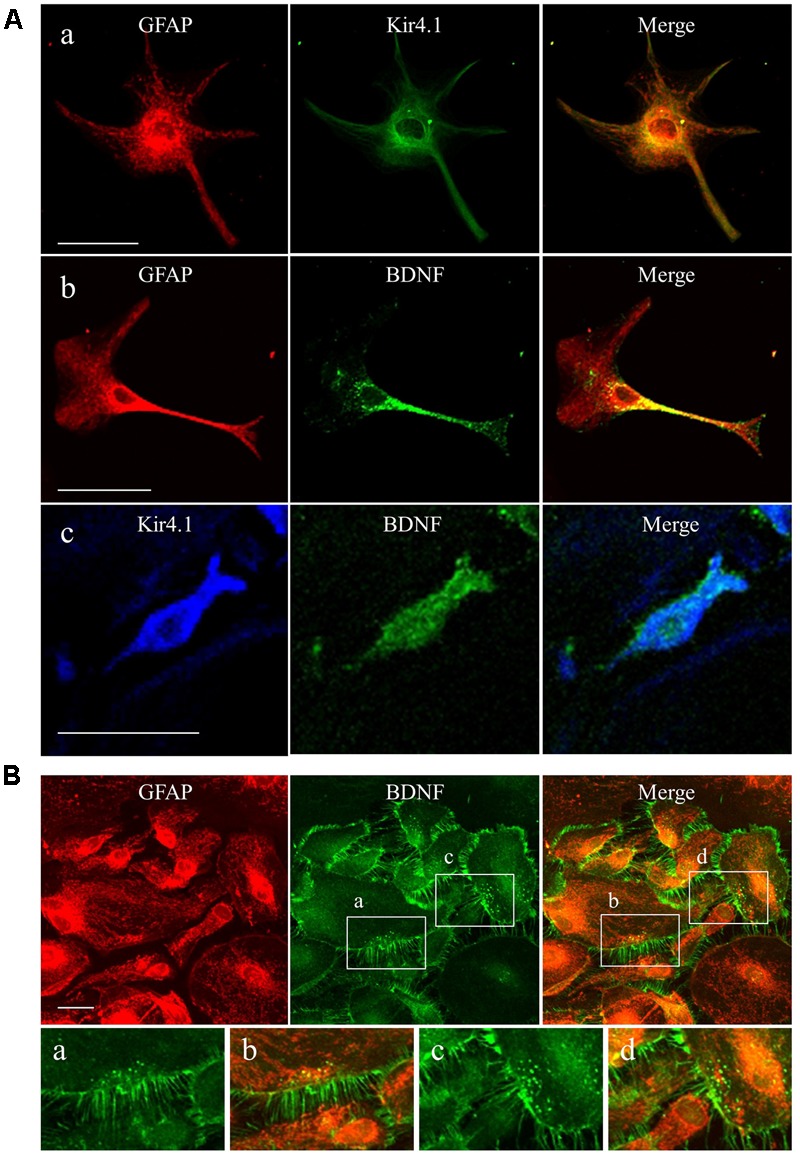
Expression patterns of Kir4.1 channels and BDNF in cultured astrocytes. **(A)** Representative images of double-immunofluorescent staining for GFAP and Kir4.1 **(a)**, GFAP and BDNF **(b)**, or Kir4.1 and BDNF **(c)** in single astrocytes. **(B)** Clustered astrocytes stained for GFAP and BDNF in culture preparations. Lower panels showed magnified images of region indicated by squares **(a–d)**. Scale bar: 50 μm (**Aa**, **Ab**, and **B**) or 25 μm **(Ac)**.

We first confirmed the expression pattern of Kir4.1 and BDNF in our astrocyte primary cultures using the immunofluorescent double staining method. Confocal laser microscopic analysis of single astrocyte revealed that Kir4.1-immnoreactivity (IR) was mostly detected in both somata and foot processes probed by the astrocyte marker GFAP (**Figure 1Aa**), resembling *in vivo* expression patterns reported previously (Harada et al., 2013; Nagao et al., 2013). A lot of granules positive for BDNF-IR, spreading from somata to end foots, were also co-stained with GFAP (**Figure 1Ab**). In addition, the BDNF-IR were detected in Kir4.1-IR positive cells (**Figure 1Ac**). In the confluent conditions, astrocytes with various cell shapes normally gathered together and formed large cell clusters (**Figure 1B**). These astrocytes possess numerous GFAP-IR negative thin processes surrounding the GFAP-IR positive somata. Interestingly, BDNF were densely expressed in these fine processes of astrocytes, reflecting a secretary process of BDNF. On the other hand, our astrocyte primary cultures did not contain NeuN (a neuron-specific marker)-IR positive cells (data not shown).

### Effects of Kir4.1 Channel Blocker on BDNF Expression in Astrocytes

**FIGURE 2:**
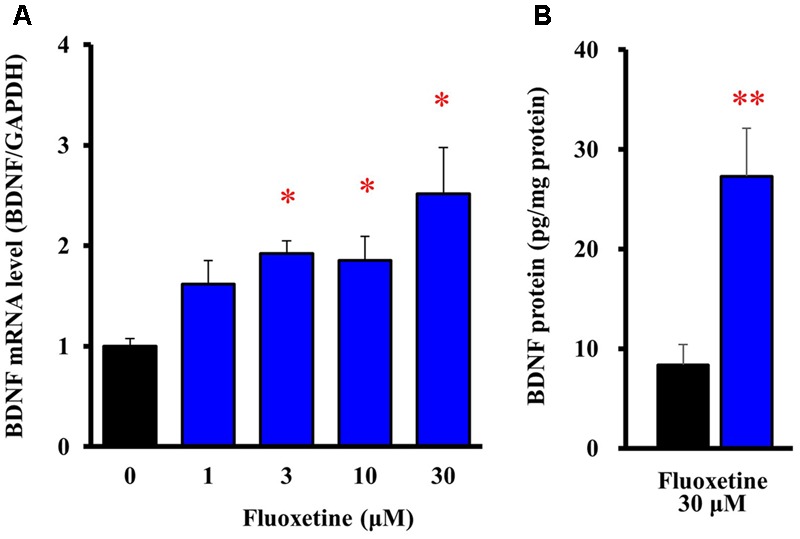
Effects of fluoxetine on BDNF expression in astrocytes. **(A)** Effects of fluoxetine on BDNF mRNA expression. Astrocytes were treated with the indicated concentrations of fluoxetine (1–30 μM) and BDNF mRNA expression was analyzed by quantitative reverse transcription (RT)-PCR. **(B)** Effects of fluoxetine on BDNF protein expression. Astrocytes treated with 30 μM fluoxetine for 24 h and the BDNF protein levels were analyzed by ELISA. The BDNF mRNA levels are expressed as the ratio to GAPDH mRNA. Each point represents the mean ± SEM of 5–6 separate experiments. ^∗^*P* < 0.05, ^∗∗^*P* < 0.01, significantly different from the astrocytes treated with vehicle.

To evaluate expressional changes in BDNF due to the Kir4.1 channel blockade, cultured astrocytes were treated with a subunit-specific Kir4.1 channel blocker, fluoxetine (see **Supplementary Figure S1**) for 8 h. Under these conditions, fluoxetine (1–30 μM) increased BDNF mRNA expression in a concentration-dependent manner and this increase reached statistical significance at 3–30 μM (**Figure 2A**). In addition, the BDNF protein levels also significantly increased in astrocytes following the treatment with 30 μM fluoxetine for 24 h (**Figure 2B**).

**FIGURE 3:**
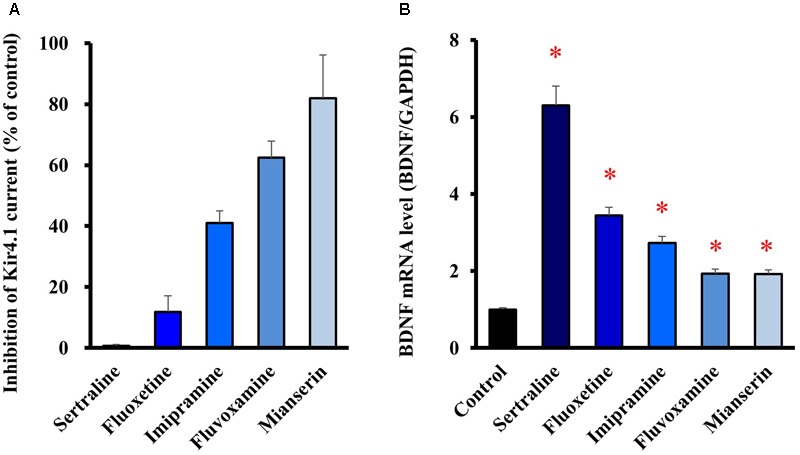
Relationship between potencies of antidepressants for the blockade of Kir4.1 channels and for the astrocytic BDNF induction. **(A)** Inhibition percentage (I_Drug_/I_Control_) of Kir4.1-conducted currents by sertraline (30 μM), fluoxetine (30 μM), imipramine (100 μM), fluvoxamine (100 μM), or mianserin (100 μM) in HEK293T cells. **(B)** Enhancement of BDNF mRNA expression by sertraline, fluoxetine, imipramine, fluvoxamine, or mianserin in astrocytes. Astrocytes were treated with each antidepressant (30 μM) for 8 h and the BDNF mRNA expression was analyzed by RT-PCR. The BDNF mRNA levels are expressed as the ratio to GAPDH mRNA. Each point represents the mean ± SEM of six separate experiments. ^∗^*P* < 0.05, significantly different from the astrocytes treated with vehicle.

We also tested several antidepressants which exhibit different blocking activities for Kir4.1 channels. When selective serotonin reuptake inhibitors (SSRIs), fluoxetine (30 μM), sertraline (30 μM) and fluvoxamine (100 μM), a tricyclic antidepressant imipramine (100 μM), and a tetracyclic antidepressant mianserin (100 μM) were applied to the Kir4.1 channel-expressing HEK293 cells, these agents inhibited Kir4.1-conducted potassium currents in the following order, sertraline > fluoxetine > imipramine > > fluvoxamine > > mianserin (**Figure 3A**). Treatment of cultured astrocytes with these agents (at 30 μM) for 8 h increased the BDNF mRNA expression with potencies as follows, sertraline > fluoxetine > imipramine > > fluvoxamine = mianserin (**Figure 3B**). Thus, there was a similarity in the relative potencies of antidepressants for the blockade of Kir4.1 channels and for the induction of BDNF mRNA in astrocytes. These drugs did not affect the cell viability of astrocytes (data not shown). In addition, it should be noted that mRNA expression of the serotonin transporter (Slc6a4) was not detected in our astrocyte preparations (data not shown).

### Effects of Kir4.1 Knockdown on BDNF Expression in Astrocytes

**FIGURE 4:**
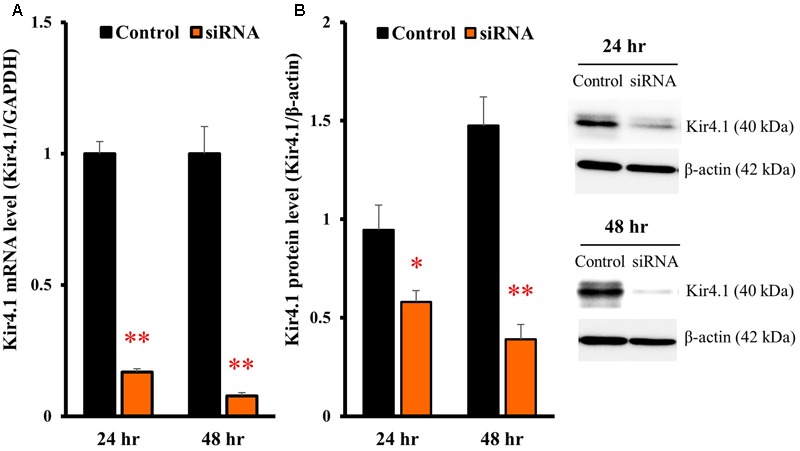
Knockdown of Kir4.1 channels in astrocytes transfected with Kir4.1 siRNA. Kir4.1 mRNA levels **(A)** and protein levels **(B)** were analyzed by RT-PCR or Western blotting, respectively, at 24 or 48 h after transfection of Kir4.1 siRNA. The Kir4.1 mRNA and protein levels are expressed as the ratio to GAPDH mRNA and β-actin protein, respectively. Each point represents the mean ± SEM of 5–6 separate experiments. ^∗^*P* < 0.05, ^∗∗^*P* < 0.01, significantly different from the negative control.

To knockdown the Kir4.1 expression, astrocytes were transfected with Kir4.1 siRNA (**Table 1**), and total RNA or protein was isolated at 24 or 48 h after the transfection. The results of quantitative reverse transcription (RT)-PCR showed that the Kir4.1 siRNA effectively suppressed the Kir4.1 expression in astrocytes, compared with the negative control, from 24 to 48 h after the transfection (**Figure 4A**). In addition, Western blotting confirmed that the Kir4.1 protein level was significantly decreased at 24 h after the transfection, more markedly at 48 h (**Figure 4B**).

**FIGURE 5:**
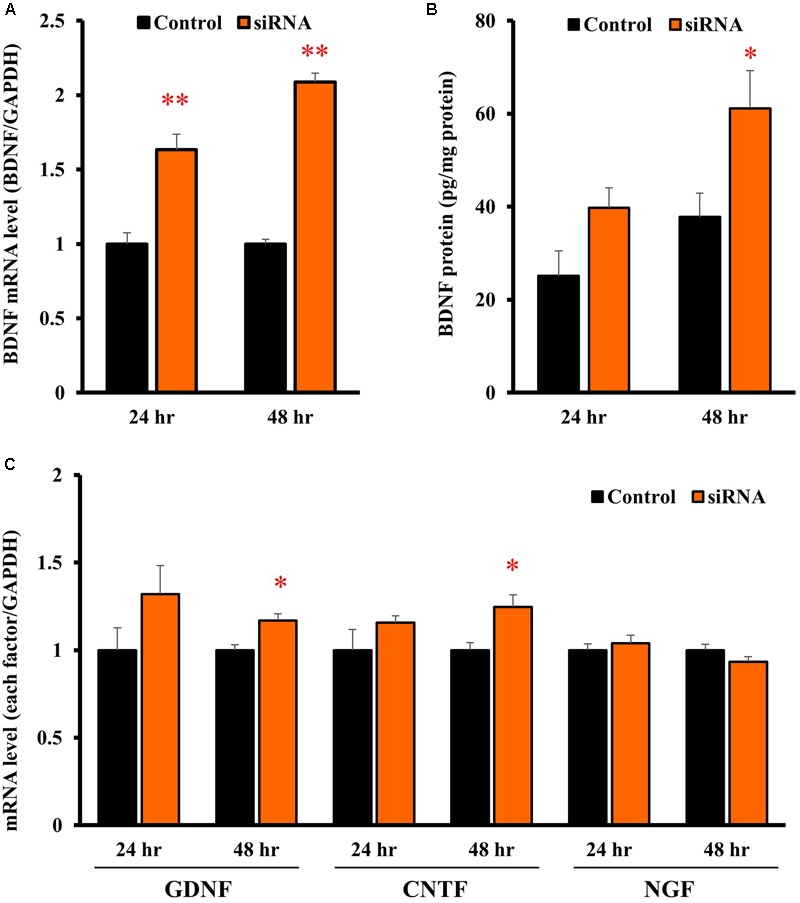
Effects of Kir4.1 knockdown on BDNF and other neurotrophic factors in astrocytes. Effects of Kir4.1 siRNA transfection on BDNF mRNA **(A)** and protein levels **(B)**. The BDNF mRNA levels and protein levels were analyzed by RT-PCR and ELISA, respectively, in astrocytes at 24 or 48 h after the transfection of Kir4.1 siRNA. **(C)** Effects of Kir4.1 siRNA transfection on mRNA levels of GDNF, CNTF, and NGF. The GDNF, CNTF, or NGF mRNA level was analyzed by RT-PCR in astrocytes at 24 or 48 h after transfection of Kir4.1 siRNA. The mRNA and protein levels are expressed as the ratio to GAPDH mRNA and β-actin protein, respectively. Each point represents the mean ± SEM of 5–6 separate experiments. ^∗^*P* < 0.05, ^∗∗^*P* < 0.01, significantly different from the negative control.

In astrocytes transfected with Kir4.1 siRNA, mRNA expression of BDNF was significantly increased at both 24 and 48 h after the transfection (**Figure 5A**). The BDNF protein levels were also increased by Kir4.1 knockdown at 24 and 48 h, and a statistical significance was obtained at 48 h (**Figure 5B**).

We also examined the effects of Kir4.1 knockdown on mRNA expression of other neurotrophic factors, GDNF, ciliary neurotrophic factor (CNTF), and NGF. Kir4.1 knockdown slightly, but significantly, increased GDNF and CNTF mRNA at 48 h. However, the mRNA expression of neither GDNF (24 h), CNTF (24 h) nor NGF was affected by Kir4.1 knockdown (**Figure 5C**).

### Signaling Pathways Regulating BDNF Expression by Kir4.1 Knockdown

**FIGURE 6:**
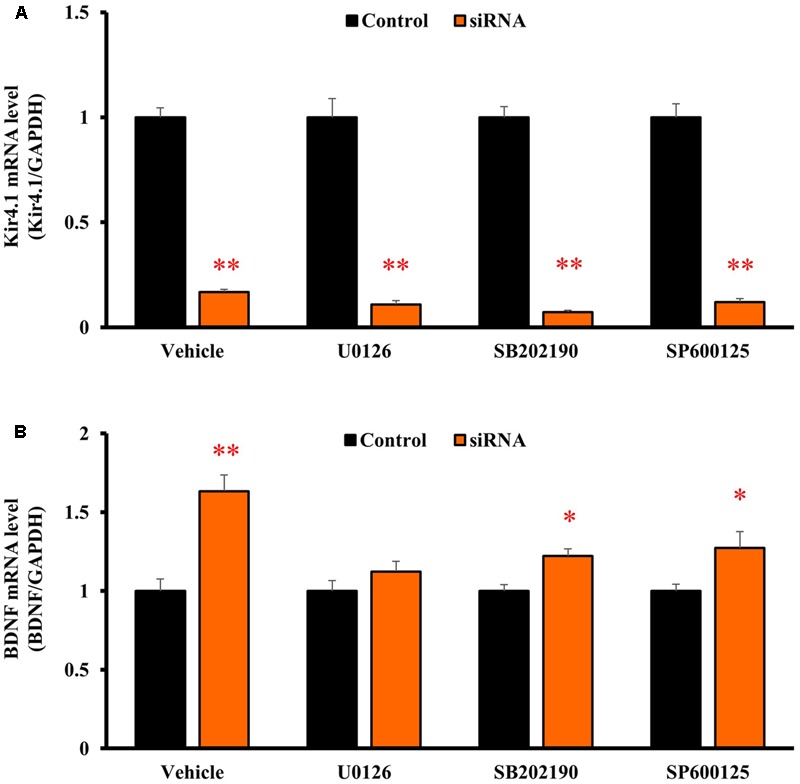
Effects of U0126, SB202190, and SP600125 on BDNF mRNA induction by Kir4.1 knockdown in astrocytes. Astrocytes were pretreated with 10 μM U0126, 10 μM SB202190, or 10 μM SP600125 for 30 min, and transfected with Kir4.1 siRNA or a negative control. The mRNA expression of Kir4.1 **(A)** or BDNF **(B)** was analyzed by RT-PCR in astrocytes at 24 h after transfection of Kir4.1 siRNA. The mRNA levels are expressed as the ratio to GAPDH mRNA. Each point represents the mean ± SEM of 5–6 separate experiments. ^∗^*P* < 0.05, ^∗∗^*P* < 0.01, significantly different from the negative control.

To clarify the signaling pathways underlying the BDNF expression changes due to Kir4.1 knockdown, astrocytes were pretreated with U0126 [a non-competitive inhibitor of mitogen-activated protein kinase (MEK) 1/2, 10 μM], SB202190 (an inhibitor of p38 MAPK, 10 μM), or SP600125 [an inhibitor of Jun amino-terminal kinase (JNK), 10 μM] for 30 min before the transfection with Kir4.1 siRNA or a negative control. These agents did not affect the Kir4.1 siRNA-induced knockdown of Kir4.1 in transfected astrocytes (**Figure 6A**). However, U0126 significantly suppressed the Kir4.1 siRNA-induced increase in BDNF mRNA expression (**Figure 6B**). In contrast, SB202190 or SP600125 did not fully inhibit the induction of BDNF mRNA expression due to Kir4.1 siRNA, whereas a slight reduction in BDNF mRNA level was observed with these agents.

## Discussion

Lack of a specific antagonist for Kir4.1 channels, as well as the early premature death of Kir4.1 knockout animals, have hampered the detailed analysis of Kir4.1 channel functions for a long time. Nonetheless, we have previously shown that several antidepressant agents, including SSRIs and tricyclic antidepressants, specifically bound to and blocked Kir4.1 channels without affecting Kir1.1 or Kir2.1 channel activities (Ohno et al., 2007; Su et al., 2007; Furutani et al., 2009). These actions were not mediated by the serotonergic system (e.g., serotonin transporters), but were due to direct and specific binding to the pore region of Kir4.1 channels. Alanine-scan mutagenesis analysis revealed that two amino acids, Thr128 and Glu158, which are located on the pore and the 2nd transmembrane (TM-2) domain of Kir4.1, respectively, were responsible for the blockade of Kir4.1 channels by the antidepressants (e.g., fluoxetine and nortriptyline). *In silico* pharmacophore-docking analysis suggested that Thr128 and Glu158, facing the central cavity of Kir4.1 channels, bind to the antidepressants with hydrogen and ionic bonds, respectively (Furutani et al., 2009; Ohno et al., 2015). By testing these Kir4.1 channel blockers, we herein demonstrated that blockade of Kir4.1 channels significantly enhanced the BDNF production in astrocytes. Although we cannot directly translate the drug actions in Kir4.1-expressing HEK293 cells to those in astrocytes, various antidepressants increased BDNF mRNA expression with relative potencies (sertraline > fluoxetine > imipramine > >fluvoxamine = mianserin) consistent with those for the inhibition of Kir4.1 currents. It seems unlikely that antidepressants-induced BDNF expression is mediated by their interactions with serotonin transporters, since their relative potencies for Kir4.1 inhibition-induced BDNF expression were different from those for the inhibition of serotonin reuptake (imipramine > fluoxetine = fluvoxamine > sertraline > > mianserin) (Mortensen et al., 2001). Indeed, mRNA expression of serotonin transporter was not detected in our cultured astrocytes, consistent with previous studies (Kong et al., 2002; Li et al., 2012). Furthermore, suppression of Kir4.1 expression by the Kir4.1 siRNA transfection also enhanced BDNF production in astrocytes. The present results therefore strongly suggest that inhibition (blockade and expressional down-regulation) of Kir4.1 channels enhances BDNF synthesis and secretion from astrocytes. Interestingly, the modulatory role of Kir4.1 channels was relatively specific for the BDNF expression and much weakly affected the expression of other neurotrophic factors although the GDNF and CNTF expression were slightly increased.

**FIGURE 7:**
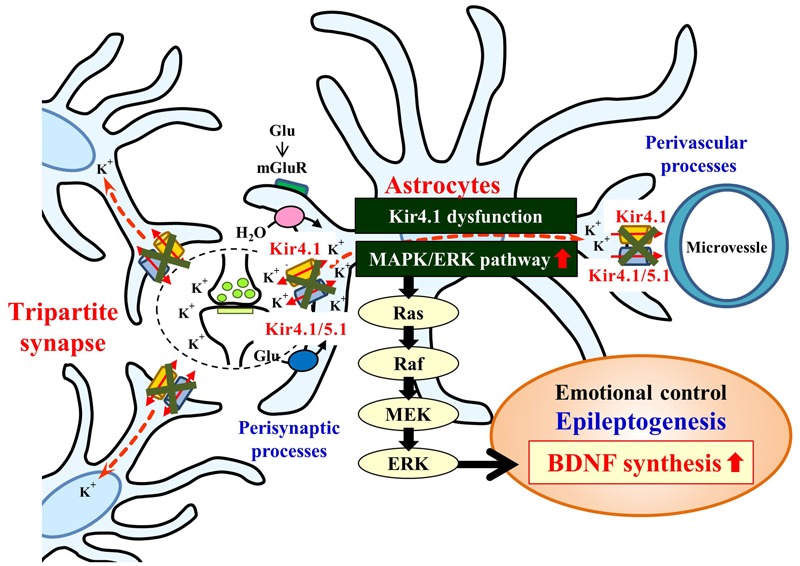
Schematic drawing illustrating the effects of the Kir4.1 inhibition on the BDNF expression in astrocytes. Inhibition (blockade and knockdown) of Kir4.1 channels activates the Ras/Raf/MEK/ERK signaling pathway and enhances BDNF expression in astrocytes, which modulates the development of epilepsy (epileptogenesis) and other neuropsychiatric disorders (e.g., major depression).

Mechanisms for the BDNF induction by Kir4.1 channel inhibition are still uncertain at this stage. In the present study, the enhancement of BDNF mRNA expression by Kir4.1 knockdown was selectively suppressed by the MEK1/2 inhibitor U0126, but not by the p38 MAPK inhibitor SB202190 or the JNK inhibitor SP600125. These results suggest that Kir4.1 knockdown-induced BDNF expression in astrocytes is primarily mediated by activation of the Ras/Raf/MEK/ERK signaling pathways (**Figure 7**). This possibility was supported by the previous findings that the Ras/Raf/MEK/ERK signaling pathways regulate the transcription of BDNF and other survival/plasticity genes through interaction with cyclic AMP response element binding protein (CREB) (Curtis and Finkbeiner, 1999; Duman and Voleti, 2012). However, we cannot completely deny a possible involvement of the p38 MAPK and/or JNK system in the BDNF induction since SB202190 and SP600125 slightly reduced the enhancement of BDNF expression by Kir4.1 knockdown.

Evidence is accumulating that dysfunction of Kir4.1 channels causes epileptic disorders in humans. The mutation of the *KCNJ10* gene encoding Kir4.1 conveys the EAST/SeSAME syndrome manifesting GTCS in the early postnatal stage (2–3 months after birth) (Bockenhauer et al., 2009; Scholl et al., 2009; Reichold et al., 2010). In addition, genetic association of the *KCNJ10* gene polymorphism or down-regulation of the Kir4.1 expression was observed in idiopathic generalized epilepsy patients (Phani et al., 2014; Guo et al., 2015) and TLE patients (Das et al., 2012; Heuser et al., 2012; Steinhäuser et al., 2012), respectively. The most frequent mutation of *KCNJ10* in patients with EAST/SeSAME syndrome was Arg65Pro at the cytoplasmic end of the TM-1 domain. Other mutations include Thr57Ile, Arg65Cys, Phe75Leu, Gly77Arg (TM-1), Phe119fs (pore helix), Cys140Arg (extracellular loop between TM-1 and TM-2), Thr164Ile, Ala167Val (cytoplasmic end of TM-2), Arg175Gln, Arg199X, Arg204His, Leu218Phe, Val259fs, and Arg297Cys (C-terminal domain) (Reichold et al., 2010; Sala-Rabanal et al., 2010; Tang et al., 2010; Freudenthal et al., 2011; Scholl et al., 2012; Kara et al., 2013; Hasan et al., 2017; Papavasiliou et al., 2017). All these mutations commonly caused suppression of Kir currents conducted by Kir4.1 channels and Kir4.1/5.1 channels, which consequently elevates the extracellular K^+^ and glutamate levels at synapses and increases neural excitability. Besides these ictogenic influences, the present study provides evidence that inhibition of Kir4.1 channels enhances BDNF expression in astrocytes. Although the mechanisms of BDNF in facilitating epileptogenesis (the development of chronic epilepsy) are still uncertain, numerous studies have shown that BDNF expression were up-regulated in various animal models of epilepsy and in human epileptic disorders and that inhibition of BDNF/TrkB function suppressed the development of epilepsy, suggesting that BDNF plays a causal role in epilepsy induction (Kokaia et al., 1995; Binder et al., 1999; Jankowsky and Patterson, 2001; Barton and Shannon, 2005; Heinrich et al., 2011; Grabenstatter et al., 2012; Liu et al., 2013). Thus, secreted BDNF facilitate epileptogenesis possibly through acting on TrkB receptors in neurons (e.g., causing neural sprouting) (Kokaia et al., 1995; Jankowsky and Patterson, 2001; Hagihara et al., 2005). In addition, since activation of truncated TrkB-T1 receptors in astrocytes have been shown to facilitate astrogliosis, this may also be involved in epileptogenesis (Goutan et al., 1998; Climent et al., 2000).

Besides epilepsy, BDNF is implicated in the pathogenesis and treatment of neuropsychiatric disorders such as major depression (Shimizu et al., 2003; Lang et al., 2004) and autism spectrum disorder patients (Sicca et al., 2011; Sicca et al., 2016). The present finding that several antidepressants enhanced the BDNF expression by inhibiting astrocytic Kir4.1 channels leads to a hypothesis that astrocytic Kir4.1 channels can serve as a novel therapeutic target for the antidepressants (Ohno and Kurachi, 2007; Ohno et al., 2015). This possibility was supported by previous findings that antidepressants induce the BDNF expression in astrocytes (Allaman et al., 2011; Hisaoka-Nakashima et al., 2016). In addition, involvement of the MEK signaling pathway in induction of BDNF by the Kir4.1 knockdown was consistent with the mechanisms reported in the antidepressants-induced BDNF induction (Hisaoka-Nakashima et al., 2016). Thus, the Kir4.1-BDNF system in astrocytes may be important as a non-monoaminergic mechanism underlying the antidepressants’ action. Since pathophysiological alterations of astrocytic Kir4.1 channels were also demonstrated in Parkinson’s disease (Howells et al., 2000; Ziebell et al., 2012), Alzheimer’s disease (Wilcock et al., 2009), amyotrophic lateral sclerosis (Kaiser et al., 2006; Bataveljić et al., 2012), and Huntington’s disease (Tong et al., 2014), further studies are required to elucidate the pathophysiological roles of the astrocytic Kir4.1-BDNF system in central nervous system disorders.

## Conclusion

We studied the effects of Kir4.1 inhibition on expression of BDNF in primary cultured astrocytes. Blockade of Kir4.1 channels by antidepressants significantly enhanced the BDNF expression. In addition, knockdown of Kir4.1 by siRNA transfection also elevated the BDNF expression, which was preferentially suppressed by the MEK1/2 inhibitor U0126. The present results strongly suggest that inhibition of Kir4.1 channels facilitates BDNF expression in astrocytes, probably by activating the Ras/Raf/MEK/ERK pathway. Thus, the Kir4.1-BDNF system in astrocytes seems to be linked at least partly to the development of epilepsy (epileptogenesis) and other neuropsychiatric disorders. However, due to the limitations associated with experiments using cultured astrocytes, further studies are required to clarify the role and mechanisms of Kir4.1 channels in regulating BDNF expression.

## Author Contributions

YO designed the research. MK, TM, YN, YM, YT, ST, KT, SS, SI, and YO performed experiments. MK, TM, SS, HI, AIk, AIn, YK, and YO analyzed data. MK, SS, HI, AIk, and YO wrote the paper.

## Conflict of Interest Statement

The authors declare that the research was conducted in the absence of any commercial or financial relationships that could be construed as a potential conflict of interest.

## Supplementary Material

FIGURE S1 Representative current traces showing inhibitory effects of fluoxetine (3–30 μM) on Kir4.1-conducted currents in HEK293T cells. HEK293T cells expressing Kir4.1 channels were voltage-clamped at -40 mV and a pair of step pulses (±70 mV, 500 ms duration with a 200-ms interval) were applied every 20 s. The arrowhead indicates the current level recorded at -40 mV.
