# Supplementary figures and images for: Inhibition of Inwardly Rectifying Potassium (Kir) 4.1 Channels Facilitates Brain-Derived Neurotrophic Factor (BDNF) Expression in Astrocytes
Source: Front Mol Neurosci. 2017 Dec 7;10:408. doi: 10.3389/fnmol.2017.00408 (PMC5768989; doi:10.3389/fnmol.2017.00408)

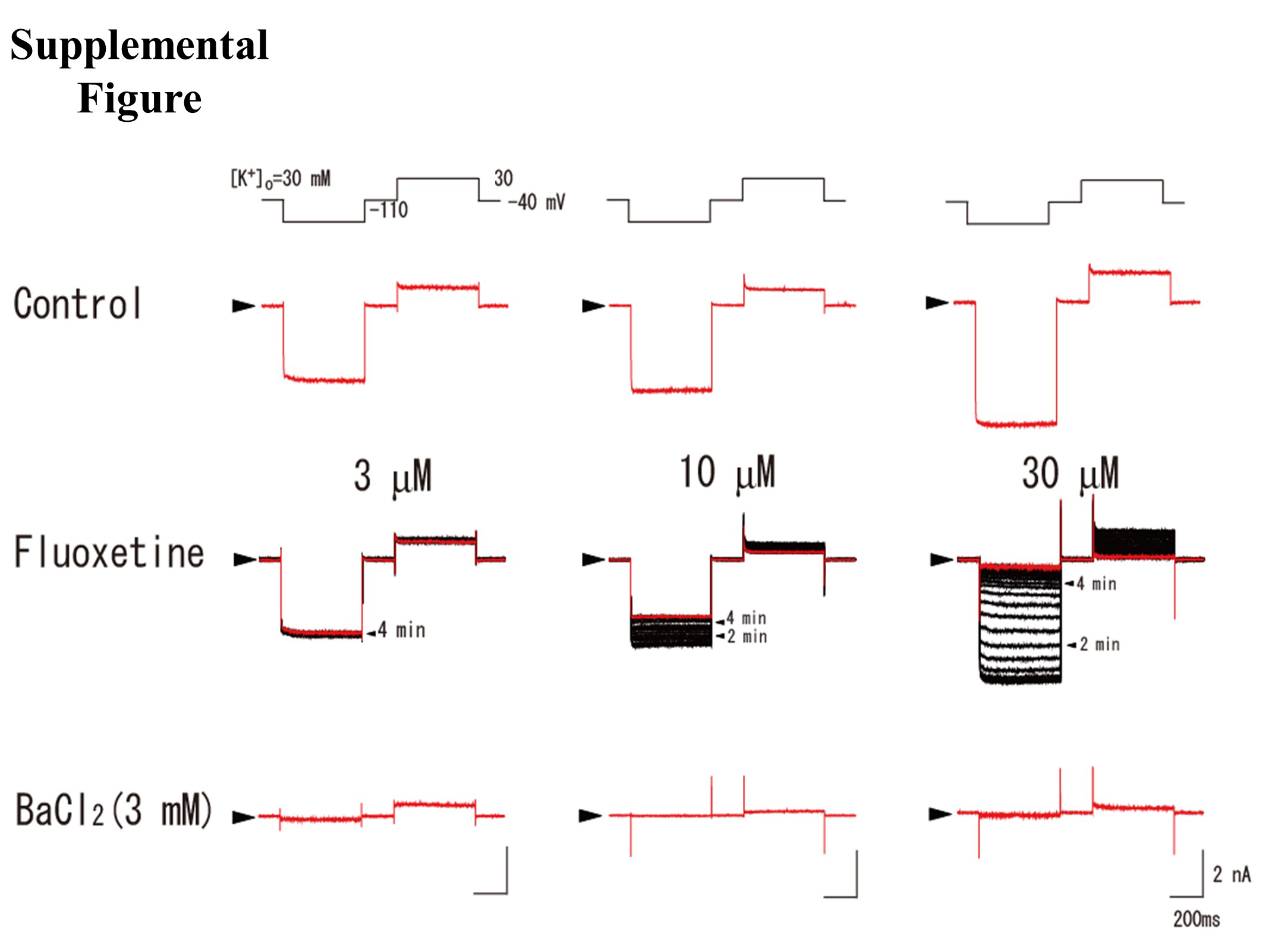

Supplement: FIGURE S1 — Representative current traces showing inhibitory effects of fluoxetine (3–30 μM) on Kir4.1-conducted currents in HEK293T cells. HEK293T cells expressing Kir4.1 channels were voltage-clamped at -40 mV and a pair of step pulses (±70 mV, 500 ms duration with a 200-ms interval) were applied every 20 s. The arrowhead indicates the current level recorded at -40 mV. [file Image_1.JPEG]
